# Sampling of the conformational landscape of small proteins with Monte Carlo methods

**DOI:** 10.1038/s41598-020-75239-7

**Published:** 2020-10-23

**Authors:** Nana Heilmann, Moritz Wolf, Mariana Kozlowska, Elaheh Sedghamiz, Julia Setzler, Martin Brieg, Wolfgang Wenzel

**Affiliations:** Institute of Nanotechnology (INT), Karlsruhe Institute of Technology (KIT), Hermann-von-Helmholtz-Platz 1, 76344 Eggenstein-Leopoldshafen, Germany

**Keywords:** Protein folding, Computer science, Computational methods

## Abstract

Computer simulation provides an increasingly realistic picture of large-scale conformational change of proteins, but investigations remain fundamentally constrained by the femtosecond timestep of molecular dynamics simulations. For this reason, many biologically interesting questions cannot be addressed using accessible state-of-the-art computational resources. Here, we report the development of an all-atom Monte Carlo approach that permits the modelling of the large-scale conformational change of proteins using standard off-the-shelf computational hardware and standard all-atom force fields. We demonstrate extensive thermodynamic characterization of the folding process of the α-helical Trp-cage, the Villin headpiece and the β-sheet WW-domain. We fully characterize the free energy landscape, transition states, energy barriers between different states, and the per-residue stability of individual amino acids over a wide temperature range. We demonstrate that a state-of-the-art intramolecular force field can be combined with an implicit solvent model to obtain a high quality of the folded structures and also discuss limitations that still remain.

## Introduction

Conformational change is one of the most prominent mechanisms of protein function and regulation^1–4^. The stability of the protein as a whole, as well as the stability of its particular conformational sub-ensembles is essential to understand and regulate protein function. Due to its dynamic nature, it is difficult to observe conformational changes directly at the single molecule level in experiment^5,6^, while computational methods, generally, struggle with the timescales involved^5,7–10^. However, the benefits of the simulation are prominent, as it provides detailed information on the mechanism of the protein folding, and identifies key intermediates and barriers to folding. In the last 20 years, there have been an enormous number of studies focused on the folding of small peptides using specialized force fields and simulation methods^11–18^, reflecting a small subsection of an active field that has evolved over decades. The universal workhorse of all these methods is the molecular dynamics (MD) method that remains constrained by the admissible timestep, which is limited by the fastest frequency of the system, i.e. typically bond-stretch vibrations in the 10^14^ Hz range^19^. At the same time, the vast majority of natural processes have a much larger inherent timescale, ranging from microseconds to seconds^20^.

To capture a single folding event, long MD simulations are required, which incurs either extreme computational cost or the need for a specialized supercomputer architecture^21^. The development of the supercomputer Anton permitted observations of many folding transitions for a range of small fast-folding proteins^22^, and even larger proteins, e.g. G-proteins^23^, using transferable biophysical force fields such as CHARMM and AMBER and explicit solvent models^24,25^. Where such hardware is not available, many strategies have been developed to circumvent the time/length scale problem by either simplifying or coarse graining the force field to accelerate the simulation protocol^26–28^, or to subdivide the simulations in many small non-equilibrium simulations^29^. MD simulations with implicit solvent also enable faster conformational sampling^30^. Indeed, the accuracy of the folding mechanism and the size of the simulated protein are limited, i.e. ~ 100 residues^27,31^, which depend on the protein complexity and the quality of the solvent model^32,33^. Some other methods, such as enhanced sampling techniques^34^, make it possible to reach longer timescales, but none of the above-mentioned approaches offer the same straightforward analysis provided by the “virtual” experiment of simulating the process as it occurs in nature^35^.

As an alternative, Monte Carlo (MC) simulation, which has no inherent timescale, has been explored as a simulation approach. MC simulations yield all thermodynamic data that can be extracted from MD simulations, and permit reconstruction of the kinetic information on long-time scales, such as folding, association or function, but do not provide direct insights into kinetics^36^. Many biological processes can be described as transitions between a few distinct conformational sub-ensembles. The barriers between these ensembles, which are directly sampled in MD, can be computed from thermodynamic averages in MC methods, which permits reconstruction of large scale kinetics even on the basis of a thermodynamic simulation^37^. In MC, special moves may be designed, which do not follow the local force, hence the change of the conformation per energy evaluation in each step may be larger, and the simulation may concentrate on a few particularly important degrees of freedom, such as dihedral angles for peptides. These advantages have the potential to accelerate molecular simulations for peptides and proteins, provided that a suitable force field can be found. Typically, in MC simulation, only a small part of the system in a single move is modulated. Efficient algorithms for multi-particles moves become more and more expensive with the number of molecules, therefore, MC with explicit solvent is particularly problematic^38,39^. Instead, implicit solvation models are well suited for MC simulations and can frequently speed up simulations by orders of magnitude. However, this is often connected to limited hydrogen bonds representation, over-stabilized salt bridges, incorrect ion distribution and neglection of the temperature dependence of the solvation free energy^31,40,41^.

Since the 90 s, the MC folding algorithms have been applied to peptides and proteins using different MC program packages developed to model aggregation and folding/unfolding behavior of peptides. Both, all-atom and coarse grained representations of proteins are used, e.g. as is known for the Rosetta model^42–44^. It implements a knowledge-guided Metropolis Monte Carlo sampling approach using a phenomenological energy function and relies heavily on the data derived from the experimental structure. Therefore, users must incorporate other biochemical information to obtain native-like models, especially for large and complex proteins. For instance, it is difficult to model and design a topology or structure that has never, or only very rarely, been observed in the Protein Data Bank^44^. PROFASI^15^, an all atom MC based C++ code, and SMMP^45^, a FORTRAN based MC code, are also known for simulation of small proteins. They are computationally fast methods and able to capture structural and thermodynamic properties of a diverse set of sequences. At the same time, coarse grained models for protein folding^46,47^, e.g. CABS (C-alpha, beta, and side chain)^48^, which uses various MC schemes, were reported with successful performance in binding studies of intrinsically disordered proteins (IDPs)^49^. By an efficient treatment of large time scale dynamics, they provide significant extension of the structural transitions and better conformational sampling while maintaining sufficient accuracy^47,50^. All atom/coarse grained multi-scale modeling techniques, such as reported by Zacharias et al.^51^ and Feig et al.^52^ (MMTSB model), applied for the scoring of the protein conformation, peptide folding and prediction of the missing protein fragments, have been also developed^52,53^. All reported MC simulations are limited to employ specifically designed force fields and algorithms, which may impact their common usage.

The improvement and the development of force fields has been an instrumental step in the advances made in protein simulation. It is therefore important to investigate whether state-of-the-art force fields, that were originally designed for explicit solvent simulations, can also be employed in accurate and predictive MC. In this context, two important questions naturally arise in the use of Monte Carlo methods: (1) will the combination of an accurate intramolecular force field, developed for all-atom MD simulations, together with the implicit solvent models yield to quantitative results, and (2) can the free-energy landscape be sampled sufficiently well, relying on simplified moves defined in MC protocols independently on the forces on the atoms?

In the following, we aim to answer these two questions by employing a Monte Carlo based protocol^54^, using an accurate implicit solvent model and a transferable all-atom intramolecular AMBER99SB*-ILDN force field^55^. This force field, in the most cases in the combination with explicit water, has been shown to perform well in mimicking experimental data using MD simulations of different peptides^24,55^. Here, we show the sampling of the conformational landscape of three conventional peptides: the 20 amino acid Trp-cage miniprotein (PDB code 1L2Y)^56^, the Villin headpiece (PDB code 1vii) comprising of 36 residues (including N-terminal methionine)^57^, and the 35-residue WW-domain (PDB code 2f21)^58^. All of the proteins belong to widely studied all-helical and β-stranded mini-proteins^42,59–62^. We focus on the reproducing of their folding free energy landscapes, barriers, and transition states in order to demonstrate thermodynamic characterization of small proteins using Monte Carlo simulations with an all-atom force field.

## Results and discussion

We investigated the conformational landscape of three, well studied, proteins of different size and tertiary structure: Trp-cage^56^, Villin headpiece^57^ and WW-domain^58^, using Monte Carlo simulations starting both from folded and unfolded structures. For each protein we sampled a wide temperature range to characterize the folding and unfolding equilibria. The computational methodology used is described in detail in section “Methods”. We start with the Trp-cage protein, being the smallest peptide investigated, and then focus on the Villin headpiece and WW-domain to show transferability and efficiency of the all-atom force field in Monte Carlo approach.

### Trp-cage protein

The Trp-cage protein (PDB 1L2Y)^56^ has been of high interest for both experimentalists and theoreticians as this short, 20 amino acid, protein is a fast folding protein (ca. 4.1 µs), enabling the introduction of different protein mutations to understand the ways of enhancing protein stability or improving drug binding efficiency (e.g., in treatment of type II diabetes mellitus)^63,64^. Moreover, this miniprotein has been used for the last two decades to benchmark force fields and modeling techniques against detailed structural, thermodynamic and kinetic data^65–74^. MC simulations of Trp-cage in the current study were performed in the temperature range of 330 K–410 K and sampling of 200 Million MC steps on the AMD EPYC 7551P node using 15 or 30 cores required for 181 h and 108 h of CPU time, respectively.

**Figure 1:**
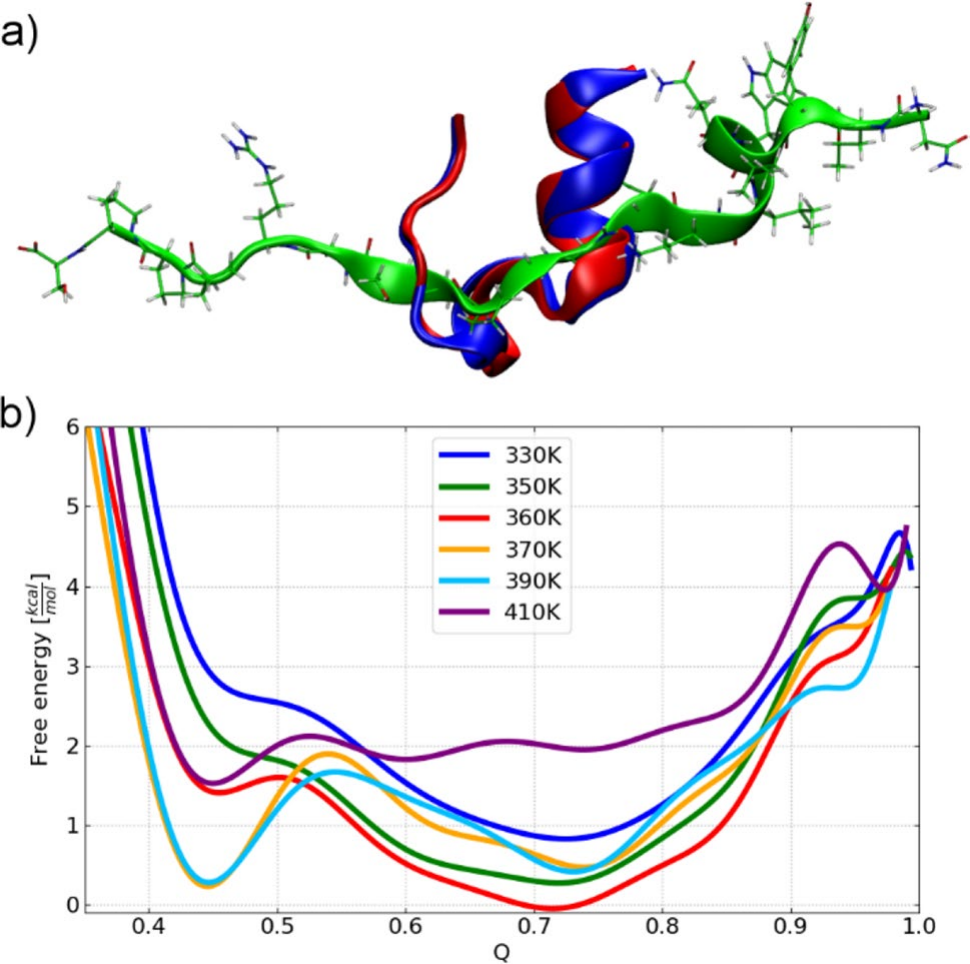
Sampling of the conformational landscape of the Trp-cage miniprotein. (**a**) Overlay of the native structure of 1L2Y (in blue) compared to the refolded structure (in red) obtained in MC simulation run at 370 K (full MC trajectory is given in Fig. S1a) started from the unfolded protein structure marked in green. (**b**) Free energy profiles as a function of the reaction coordinate Q (fraction of native contacts) at different temperatures computed from the Monte Carlo simulations. Refolded and intermediate ensembles were found at Q ~ 0.73 and 0.45, respectively. Visualization was done in VMD (version 1.9.2beta1) https://www.ks.uiuc.edu/Research/vmd/.

Trp-cage consists of a short α-helix between residues 2–9, a single turn of 3_10_-helix (residues 11–14), and a hydrophobic core made of proline residues (Pro-12, Pro-18, Pro-19) and Tyr-3, Trp-6 (see the structure in blue in Fig. 1a). Its folding is known to be modulated by cooperative interactions between water molecules and polar groups of a protein, therefore, proper treatment of the solvation environment is essential to get the correct folding behavior^75–77^. Indeed, several implicit solvent models, used in all-atom MD simulations, have been reported to yield correct refolded structures of Trp-cage^78–80^. Here, we use the AMBER99SB*-ILDN force field with the generalized Born based implicit solvent model with a solvent accessible surface area term for nonpolar solvation effects (see “Methods”). Figure 1a depicts the overlay of the native (in blue) and the refolded (in red) structure of 1L2 after its full unfolding (in green). The respective MC simulation run was performed at 370 K (see Fig. S1a) starting from the unfolded Trp-cage with the fraction of native contacts of 0.07. The refolded structures match good with the native Trp-cage (Cα-RMSD of the refolded protein, depicted in red in Fig. 1, is of 0.86 Å) with small deviations around the helix turn. This shows high quality of the force field and the sampling efficiency of the MC algorithm with the accumulated acceptance ratio of 60%.

To establish the folding temperature of the protein, the free energy profile was calculated using the potential of mean force (PMF) projected on the fraction of native contacts, Q. This measure is a widely used reaction coordinate for the folding process^77,81^ (see Fig. 1b). It reflects the similarity between the native and the predicted structure of the protein, i.e. Q ~ 0.9–1.0 is the closest to the native structure obtained in NMR. Two main states of the conformational ensemble describing its folded (Q ~ 0.73) and unfolded states were observed. We also find a partially unfolded state (Q ~ 0.45) with an energy barrier of 1.20 and 0.80 kcal·mol^−1^ at 370 K and 390 K, respectively. Similar states of Trp-cage with the free energy barrier of 0.80 kcal·mol^−1^ were reported by Zhou^82^ using highly parallel replica exchange MD with explicit solvation and in experiment^83^. We note that the primary minimum at Q ~ 0.73 is rather broad, which results from the relatively weak stabilization of the native structure in this small protein because of the implicit solvation. This was overcome in MD with explicit water, where Q ~ 0.9–1.0 of the folded Trp-cage was reported^75^. Nevertheless, the absolute value of the Q at the minimum depends on the details of the definition of the native contacts taken from the NMR ensemble.

**Figure 2:**
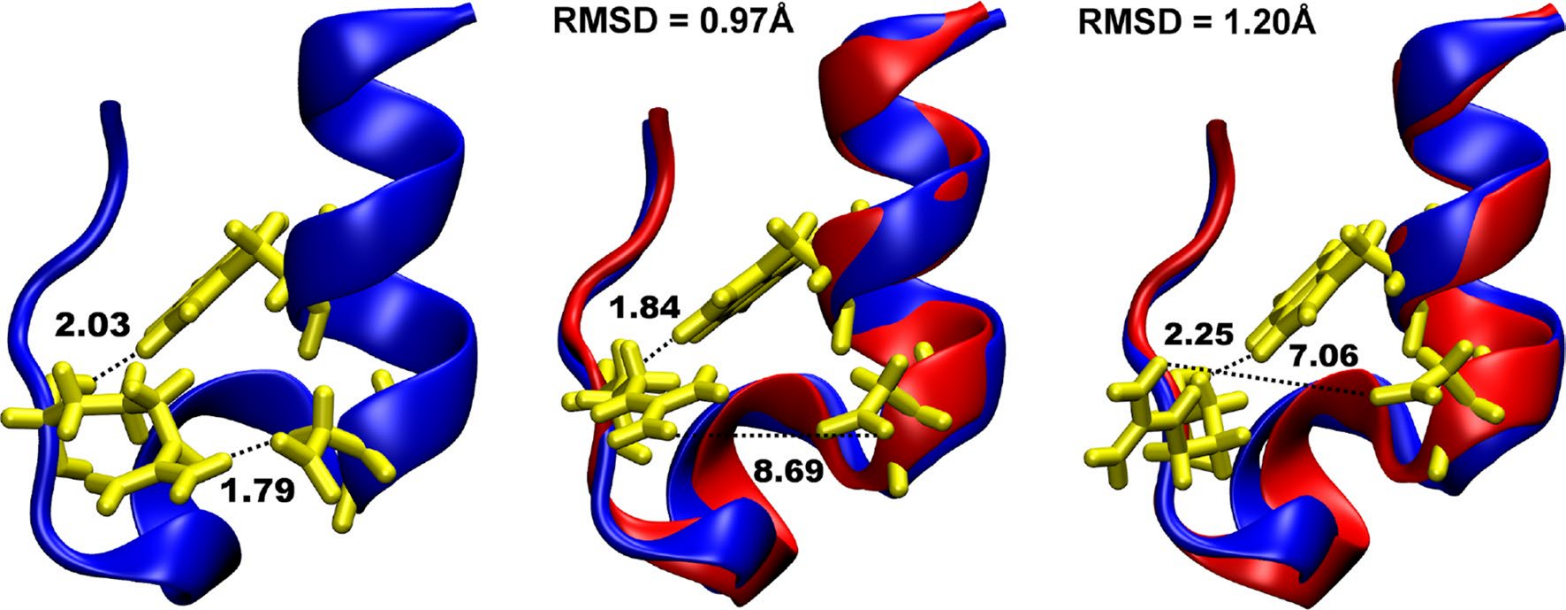
Local minima representing refolded conformers of the Trp-cage in MC simulation started from the unfolded protein structure with Q = 0.07 at 370 K (see Fig. S1a). The native structure is in blue, while refolded representatives are in red. Asp-9 and Arg-16 residues forming hydrogen-bonded salt bridge (with the length in the native state of 1.79 Å) and Trp-6 forming hydrogen-bond with Arg-16 (with the length in the native state of 2.03 Å) are colored in yellow. Visualization was done in VMD (version 1.9.2beta1) https://www.ks.uiuc.edu/Research/vmd/).

We also observe the broad range of the folding temperature of the protein, starting from 370 K. At low temperature, i.e. 330 K, we observe essentially only a single minimum, which gives way to a free energy surface with two minima around 350 K. The estimated folding temperature, i.e. the temperature when both minima are equally probable, is significantly higher than was experimentally observed, i.e. 315–317 K^64,69^, or calculated using all-atom force fields with explicit solvation, i.e. 321–326 K^76,84^, but it is in line with the folding temperatures obtained using implicit solvent models (375–400 K)^85,86^. This may result from the lack of the temperature dependence of the implicit solvent model^31^. The generalized Born solvent-accessible surface area (GBSA)-type implicit solvation models, used here, are known to over-stabilize the folded states of proteins, especially those stabilized by solvent-exposed salt bridges^85,87,88^. The breaking/formation of such H-bonded salt bridge, formed between Asp-9 and Arg-16 (N–H$\cdots$O bond of 1.79 Å in Fig. 2) outside of the central hydrophobic core region exposed to the solvent, regulates Trp-cage folding and refolding, inducing the observed increase in the folding temperature.

A detailed analysis of the refolded Trp-cage demonstrates the accuracy of the force field and MC approach with respect to structure. Several refolded structures are depicted in Figs. 2 and S2. The Cα-RMSD of the refolded protein is 0.97 Å with the average Cα-RMSD deviation during the MC run, started from the unfolded structure (shown in green in Fig. 1a), is 1.96 (0.44) Å. Secondary structure of both, α-helix and proline-end, are reproduced correctly in the refolded protein (see Fig. 2) with backbone RMSD of 0.73 Å and 0.47 Å, respectively. This is connected to the helical propensity of the helical domain of Trp-cage, which was shown to be stable even at 400 K in MD simulations with explicit water, which results from the force field parameterization that is largely helix-based^89^. Among the most flexible residues in the refolded protein, marked in Fig. S2, is Arg16, which participates in the salt bridge formation of the Trp-cage. This results in the lack of salt bridge formation in the refolded structures, as shown in Fig. 2, where the H⋯O distance between Asp-9 and Arg-16 was found to be far larger than the native one. This result probably originates from the drawbacks of the implicit solvation model, as mentioned above. Even though the salt bridge is unstable in our MC simulations, the refolded structures of Trp-cage conserve the two main secondary elements, with the most notable difference in a 3_10_-helix-like turn, and form the tertiary structure due to the stability of other H-bonding interactions present in Trp-cage. Among them is a N–H⋯O hydrogen bond between Trp-6 (H-bond donor) and Arg-16 (H-bond acceptor) with the length of 2.03 Å in the NMR-structure (marked in Fig. 2), which is stable in the refolded Trp-cage (see Fig. S2). Together with the salt bridge between Asp-9 and Arg-16, this H-bond regulates fast folding of Trp-cage^78,89^.

### Villin headpiece

**Figure 3:**
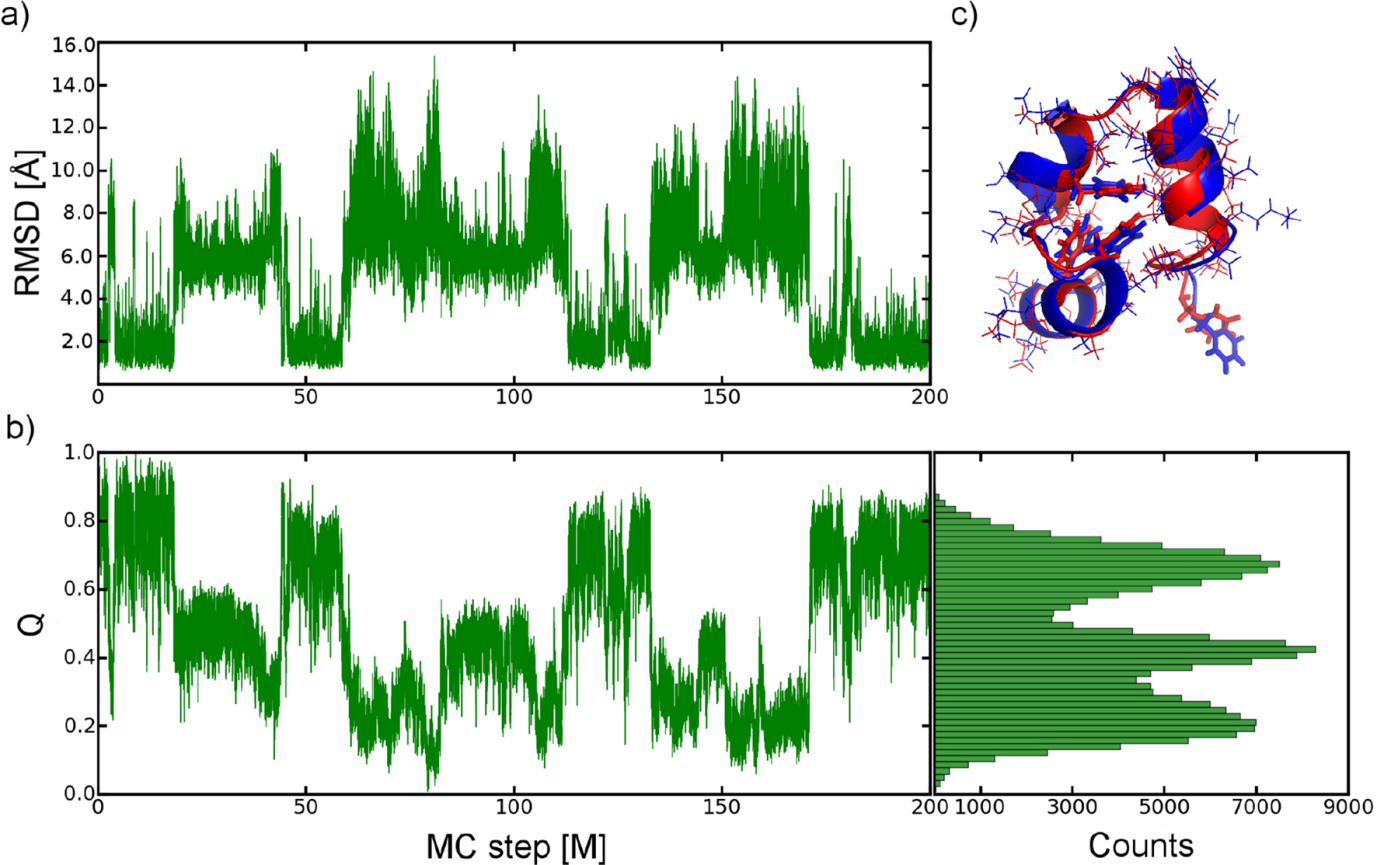
Section of a trajectory of the Villin headpiece simulations started from the native 1VII at 360 K (including temperature shift shown in Fig. S4): (**a**,**b**) RMSD and fraction of the native contacts, Q, as a function of step-size showing many transitions between well-defined native and non-native ensembles. At the right side in (**b**), the occupancy of the sub-ensembles, corresponding to the folded, intermediate and unfolded conformations are shown. (**c**) Overlay of the refolded (in red, Q = 0.8) and the experimental (in blue) conformation of the protein. Visualization was done in VMD (version 1.9.2beta1) https://www.ks.uiuc.edu/Research/vmd/.

Next, we generated multiple long MC trajectories of the Villin headpiece, known as HP-36^57^: the smallest autonomously folded protein without disulfide bonds, oligomerization or stabilizing ligands, at a wide range of temperatures (360–460 K). The Villin headpiece consists of three helices: (i) between residues 4–8, (ii) 15–18 and (iii) 23–30, as depicted in Fig. 3c. Loop, turn and a closely packed hydrophobic core held the helices together in a compact structure. Most MD simulations observe one and/or two, two-phase, folding pathways of HP-36, where helix2 serves as the structural starter of the Villin folding through intermediate and transition states to the folded structure^8^. Recently, Wang et al. have shown the third folding pathway, which starts from the hydrophobic core or/and helix3^90^.

The main aim of our analysis was to predict the near-native refolded structure of Villin using efficient sampling of its conformational space performing MC simulations with all-atom force field. Our MC algorithm results in the multiple transitions between different states of the protein (see Fig. 3a,b), enabling quantitative prediction of the protein thermodynamics. The quality of the refolded structures is good, we find an all-atom/backbone RMSD of 1.49 Å/0.76 Å after refolding to the NMR structure^57^, and unfolded conformations with RMSD > 12 Å (all-atom/backbone RMSD) are observed (see Fig. 3a). Excluding the first three unstructured residues, the refolded conformations agree to an all-atom/backbone RMSD of 1.12 Å/0.46 Å. In the high RMSD ensemble, all elements of the tertiary structure and significant fractions of the secondary structure are lost. Refolded conformations, see Fig. 3c, completely recover the native secondary and tertiary structure.

**Figure 4:**
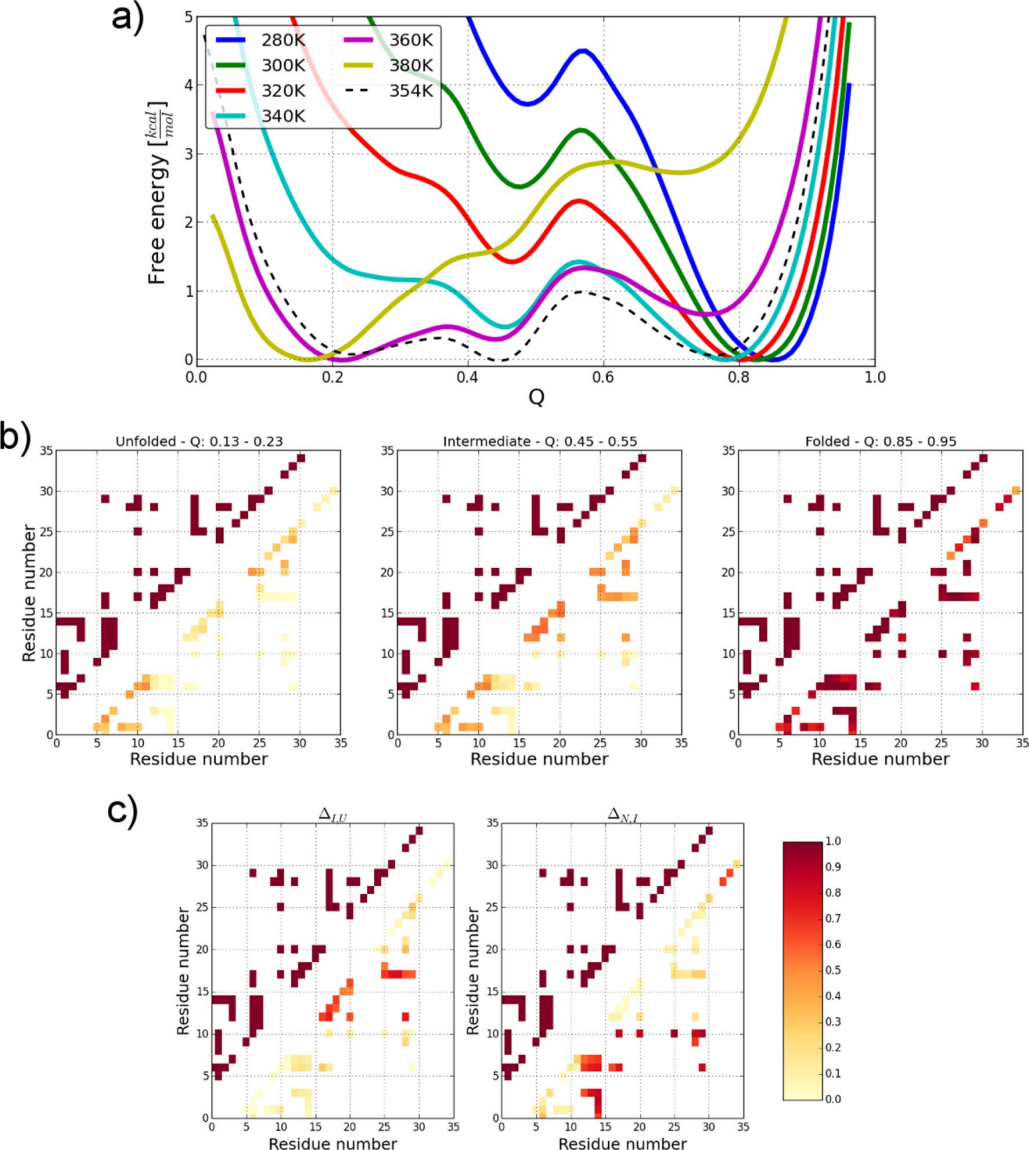
Analysis of conformational landscape of the Villin headpiece. (**a**) Free energy profiles as a function of the reaction coordinate Q at different temperatures and at the interpolated folding temperature (marked with dashed line). Native, intermediate, and unfolded ensembles were found at Q ~ 0.8, 0.45, 0.2, respectively. (**b**) Contact maps of the unfolded, intermediate, and folded ensemble (left to right) weighted by the occurrence in the ensemble (bottom right half of the figures) in comparison to the native contacts derived from the NMR structure (top left parts of the figures). (**c**) Contact difference maps showing the changes in secondary and tertiary contacts in the unfolded-intermediate transition and intermediate-native transition.

Similarly to the longest, presently available, molecular dynamics simulations^21,22,90,91^, in all simulations we observe multiple folding and unfolding events, which we use to extract thermodynamic information via the PMF projected on the fraction of the native contacts, Q (Fig. 3b). The free energy profile obtained, depicted in Fig. 4a, features three distinct minima corresponding to the native ensemble N (Q ~ 0.8), the denatured ensemble D (Q ~ 0.2) and a folding intermediate I (Q ~ 0.45). The existence of intermediate conformations is difficult to observe both in experiment and theory, but they have been clearly identified through kinetic analysis^91^.

To demonstrate the shift of the folding temperature as the result of the implicit solvation, as we have shown also for Trp-cage, we computed the specific heat capacity of the HP-36 as a function of temperature (see Fig. S4) and performed temperature calibration of the MC simulations by 83 K. In such a way, we established the folding temperature of the Villin headpiece as 354 K (see Fig. 4a), which is only 12 K shifted in comparison to the experimental value (342 K)^57^.

At this temperature, the native state is stabilized by the energy barrier of ∆G_N,I_ = 1 kcal·mol^−1^, what is in agreement with the experimental data and all-atom simulations using the same intramolecular force field and explicit water^8,20,91,92^. We also observe smaller energy barrier between the intermediate and the denatured state, i.e. ∆G_I,N_ = 0.4 kcal·mol^−1^ (as also known from experiment)^91^.

To better understand the most significant structural changes and residues responsible for the protein folding, we performed the detailed analysis of each of the three ensembles extracted from the trajectories. In Fig. 4b, the contact maps of the unfolded, intermediate, and folded ensemble are given, while in panel in Fig. 4c, the differences between the unfolded and intermediate, as well as, the intermediate and folded ensembles are demonstrated. In the folded ensemble (last panel in Fig. 4b, refolded structure with the lowest Cα-RMSD), we see a good agreement between the contacts of the folded ensemble in the simulation and the native contacts derived from the NMR structure. The unfolded ensemble shows residual secondary structure with significantly decreased probability, in comparison to the other ensembles, and essentially no tertiary contacts. As the fraction of native contacts increases, helix2 forms, but few tertiary contacts are present. This is better seen in the difference maps between the unfolded and the intermediate (left panel in Fig. 4c) and the intermediate and folded ensemble (right panel in Fig. 4c). There, large changes in the stability of tertiary contacts finally lead to the stabilization of the native conformation.

**Figure 5:**
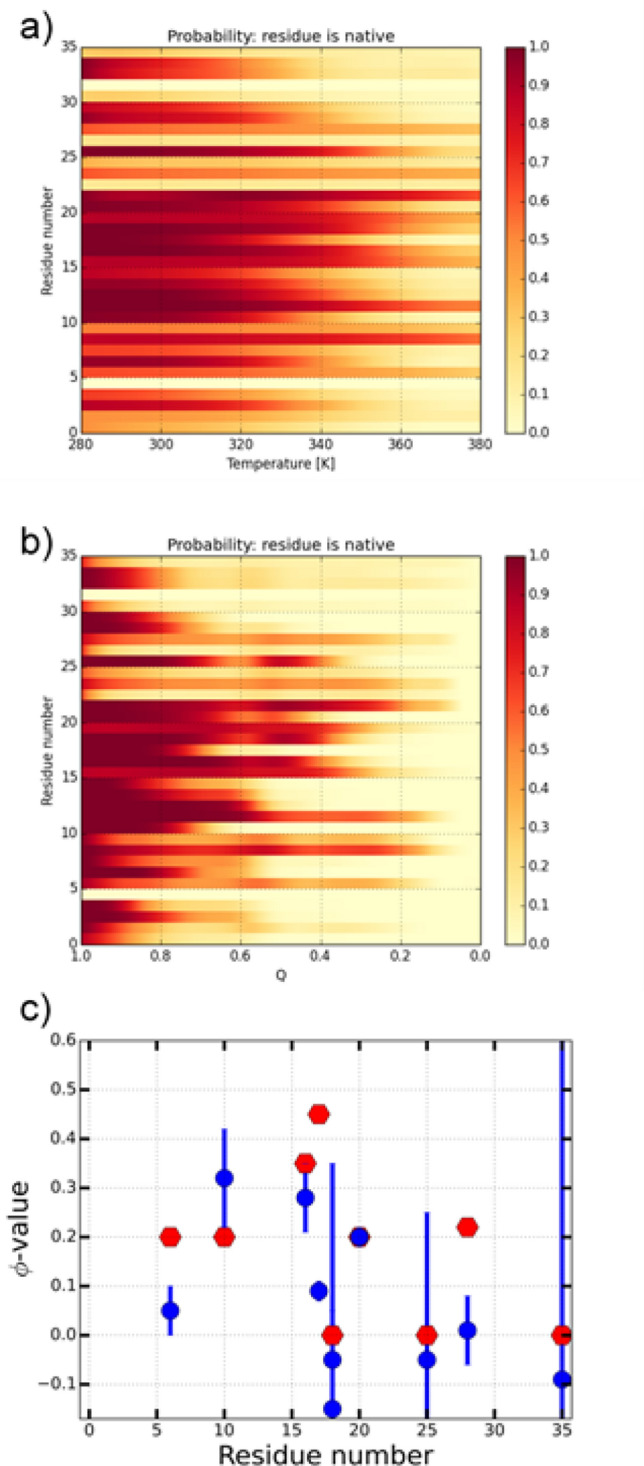
Thermal stability of Villin headpiece from MC simulations. (**a**) Probability the residue is in its native environment as a function of temperature. (**b**) Probability the residue is in its native environment as a function of established native contacts Q. White horizontal stripes in the data occur for residues that have few native contacts. (**c**) *ϕ*-values at the folding temperature, obtained from the Boltzmann-weighted fraction of native contacts for each residue for all ensembles at and close to the transition state barrier. Values in blue correspond to experimental values at 310 K^70^, red symbols result from the simulation.

The stability of the individual residues, measured by the presence of local contacts, as a function of temperature (Fig. 5a), offers a view on the parts of the protein relevant for protein folding and their thermal stability with detailed structural information on protein folding transition states. The complementary per-residue stability of individual residues at the transition temperature of 354 K, as a function of the reaction coordinate Q, is shown in Fig. 5b. There, the transition state ensemble near Q ~ 0.6 is clearly visible as a light vertical band, where native contacts are diminished partially reforming in the intermediate state. Identification of the transition state ensemble between the intermediate and the native conformation permits a computation of the *ϕ*-values, which are the measure of the presence of native contacts in the transition state. *ϕ*-values are descriptors widely used as experimental perturbation (mutation) to probe the free energy landscape. They are also used to check on simulation accuracy^93^. As shown in Fig. 5c, the computed *ϕ*-values using our approach (marked in red), show good agreement with experimental observations^91^. Combining the data obtained, the contribution of individual amino acids to protein stability is analyzed in detail: Residue Ala-18, for example, is found to be thermally highly stable (averaged over all conformations, Fig. 5a), but diminishes significantly in its native environment in the transition state (light band at Q ~ 0.6 in Fig. 5b), in comparison to both, the native and intermediate ensemble, where its *ϕ*-value is correspondingly low.

**Figure 6:**
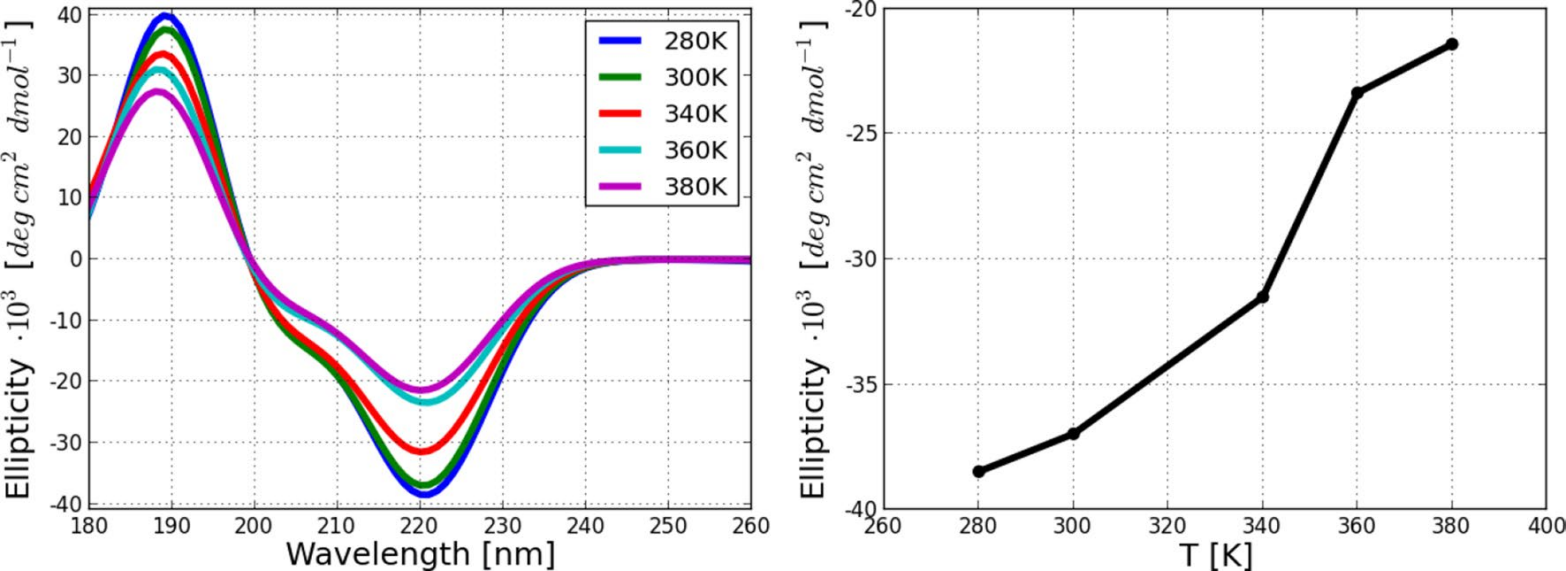
Calculated circular dichroism (CD) spectra of Villin headpiece. Left panel: Ellipticity as a function of wavelength at different temperatures of MC simulation. Right panel: ellipticity at 220 nm as a function of temperature.

The folding equilibrium at a wide range of temperatures can be also characterized by investigating the helical content of a protein via the circular dichroism (CD) spectroscopy^94^. In order to directly compare our data with experiment, we have computed the ellipticity of Villin headpiece, shown in Fig. 6 (see section “Methods” for details). Strong peaks in all three α-helical bands at 190 nm, 208 nm and 220 nm have been found. The signals weaken as a function of temperature, confirming denaturation of the protein, being in a good agreement with experimental measurements^57,91^ and MD simulations^92^. The temperature dependence of ellipticity as a function of temperature (panel on the right in Fig. 6) demonstrates the ellipticity decrease with temperature increase since all three helices lose their stability.

### WW domain

**Figure 7:**
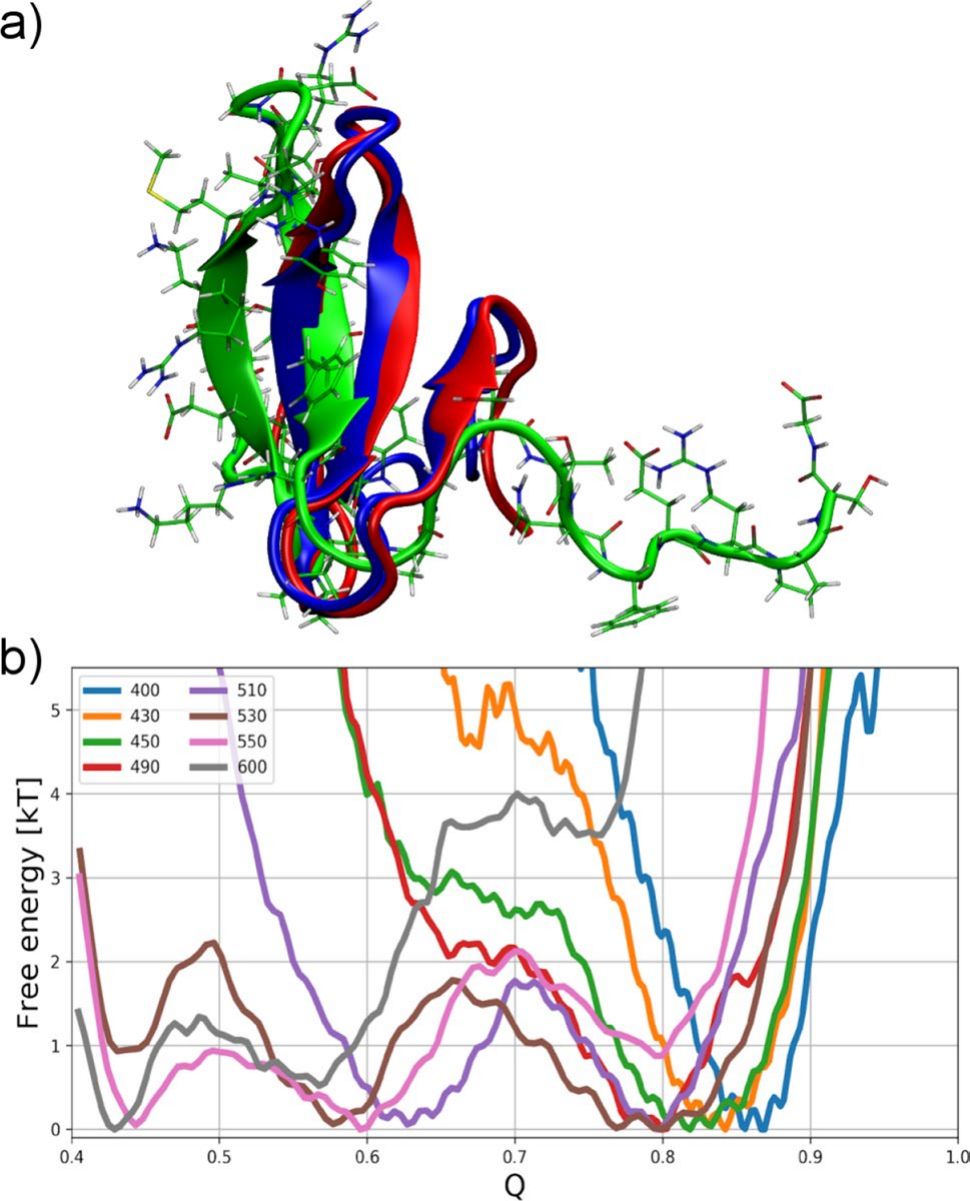
Conformational space of the WW domain in the MC simulations started from the native structure (see Fig. S6). (**a**) Representative structures of the intermediate energy minimum at Q = 0.44 (in green), refolded protein at Q = 0.80 (in red) compared to the native structure (in blue). Other refolded local minima are depicted in Fig. S5. (**b**) The free energy landscape of the WW domain mutant for different temperatures as a function of the fraction of established native contacts Q computed from the Monte Carlo simulations. Visualization was done in VMD (version 1.9.2beta1) https://www.ks.uiuc.edu/Research/vmd/.

We have observed that the folding of both Trp-cage and Villin headpiece, which are alpha-helical proteins, was in good agreement with the native structures. As a third example, we investigated the folding of a β-sheet-containing protein, i.e. the WW-domain, shown in blue in Fig. 7a. The WW domain is often used as a model protein in the investigation of β-sheet folding^58,95,96^. We performed folding simulations of the GTT mutant of the WW-domain^97^ in the regime 1 > Q > 0.4 at the temperature range of 400–600 K (every 20 K), which allows enhanced sampling of the refolding events between the native and the intermediate configurations. In most cases, the outer strands of the WW-domain unfold, while the core stays intact. We restrained the Q range for this protein because we observed that simulations that reached Q < 0.4 did not return to Q > 0.4 within the allocated computational time. This may be related to the fact that for Q < 0.4 most of the β-sheet character is lost and the nucleation of β-sheets from random coil structures is a rare event. In the following, we therefore confine the investigation to the refolding of the native configuration from partially unfolded configurations which retain some β-sheets (see below). The issue of nucleation of β-sheets from random coils has been addressed elsewhere^98^. It is known that the β-sheet nucleus is stabilized by a solvent exposed N–H⋯O hydrogen bond between Ser-13 to Arg-17 (see Fig. S7) which may be difficult to stabilize on its own in an implicit solvation model^99^. It has also been reported that implicit solvation models struggle with a correct description of the transition state of WW domain^100^. To overcome this limitation, we confined the simulation to the window in Q reported above, corresponding to the intermediate ensemble through implemented a repulsive potential in Q that drives the simulations back to Q > 0.4.

We performed MC simulations for a set of different temperatures to estimate the equilibrium folding temperature. The eight resulting free energy landscapes are shown in Fig. 7b. The minimum of the free energy surface with the highest fraction of established native contacts (Q ~ 0.80–0.85) corresponds to the global free energy minimum at low temperatures. By increasing the temperature up to 510 K, a second minimum is observed at Q ~ 0.6, where the outer two strands of the WW domain detach from the protein core.

A further minimum in the free energy landscape of the protein, i.e. at Q ~ 0.45, is observed at around 530 K. At higher temperatures, the protein is more prone to stay in the unfolded state. The simulations at 530–550 K result in multiple partial unfolding and refolding of the WW domain even during Monte Carlo simulations with 64 million MC steps (see Fig. S6). The refolded structures of the protein, for example at 530 K, with the fraction of the native contacts of Q ~ 0.8 (see Fig. 7b), are in a good agreement with the reported crystal structure of WW domain (PDB code 2f21)^80^ with the Cα-RMSD < 2.0 Å, as shown in Fig. 7a and S5. The equilibrium folding temperature was found at 530 K, again higher than known from experiment (345 K) and MD simulations (395 K)^98,101^. At the same time, we observe the free energy barriers of folding up to 2kBT (see Fig. 7b), i.e. up to 2.1 kcal·mol^-1^ at 530 K, similarly as was reported by Shaw et al.^22^.

## Discussion

In summary, we have found that the combination of a state-of-the-art intramolecular forcefield with an accurate implementation of an implicit, physics-based solvent model leads to reproducible refolding of the Trp-cage, Villin headpiece and WW domain proteins. While the refolded structures are in good agreement with the experimental data, in comparison to other MC predictions, and can be recovered from unfolded configurations for helical proteins and partially unfolded β-stranded structures, the folding temperatures are systematically overestimated. Our data show that using off-the-shelf computational hardware and the combination of the intramolecular part of the all-atom AMBER99ILDN* force field with an implicit solvent model can characterize the relevant states of helical proteins with sufficient accuracy. The reasons for the deviation in the folding temperatures are presently unclear. While the implicit solvent model has no temperature dependence, it should perform well near room temperature where most of these proteins fold. An overestimation of the folding temperature in simulation^85–88^ with an intramolecular force field that performs better in explicit solvent, means that the entropy of the unfolded configurations is underestimated. Implementation of other force fields, e.g. CHARMM with CMAP correction may improve dynamical and structural properties of proteins in their unfolded state, thus, increase the quality of MC sampled configurations^102^. Further studies will consider also the folding behavior of large proteins, which may differ significantly from those studied here.

One remaining problem is therefore the accuracy of the implicit solvent model that lacks temperature dependence and the proper description of the solvent exposed hydrogen bonding, which may lead to folding at the elevated temperatures^103^. Moreover, the differences may arise from the imbalance of the intramolecular energies of the force field calibrated for explicit water simulations and the implicit solvent model. The use of the recently reported implicit solvent models, e.g. ff14SBonlysc + GB-Neck2^31,33,104^, where advanced fitting of GB solvation energies and the relative solvation energies to Poisson-Boltzmann method for a set of proteins and peptides has been made, should be tested. Improvement in the accuracy of the solvation energies and effective radii may result in better agreement of conformational sampling in comparison to MD with explicit solvation than in GBSA. Moreover, the computational cost of more advanced models like the three dimensional reference interaction site implicit solvent model (3D-RISM)^105^, especially with the closure relation proposed by Kovalenko and Hirata (3D-RISM-KH)^105–108^, need to be considered. The latest version of this approach operates with the solvent representation by the spatial distributions of the solvent molecules around a solute macromolecule, therefore results in better solvation structure of a protein and its thermodynamics. To our knowledge, the accuracy of the 3D-RISM-KH was demonstrated for the folding of the miniprotein 1L2Y and protein G^106^. Further investigations regarding the efficiency and accuracy of implicit solvent models are needed, which further improve the MC approach using the standard off-the-shelf computational hardware and standard all-atom force fields as demonstrated in the present report.

## Conclusions

We have demonstrated that Monte Carlo simulations make it possible to efficiently sample the conformational landscape of the folding of small proteins using standard hardware without the need of extreme high-performance computing. There are two contributing factors that make the reported simulations fast. First: the efficient implementation of an implicit solvent model that significantly reduces the number of degrees of freedom (presently MC simulations are not feasible in explicit water, because there are no efficient collective moves for all the water coordinates). Secondly: acceleration by the usage of the Monte Carlo algorithm with its large effective time step, i.e. near the folding equilibrium temperature, the trajectories show a transition approximately every 5 × 10^7^ energy evaluations for the Villin headpiece. Correlating this with the experimental folding time, a single MC step covers the same distance in conformational space as an MD simulation of 5 × 10^−13^ s. The “time step” in MC is, thus, about two orders of magnitude larger than the typical MD timestep. More improvement of the current model is needed, including implementation of the algorithm for GPU acceleration.

## Methods

### Force field

The simulations were performed with the AMBER99SB*-ILDN force field^40^ and an implicit solvent model consisting of a generalized Born (GB)^80,109^ term that models polar solvation effects and a solvent accessible surface area (SASA)^110^ term that models nonpolar solvation effects. The Born radii were computed with the PowerBorn method^111^ and the SASA with the PowerSASA method^97^. The dielectric constant of the protein was taken to be *ε*_*p*_ = *1* and that of water *ε*_*w*_ = *80*. The surface tension of the nonpolar solvation term was *γ* = *5.42* cal/mol·Å^2^. No long-range cutoffs or approximate methods were used in the evaluation of the force field or implicit solvent model. In Monte Carlo simulations sometimes, large moves on atoms or groups of atoms are proposed that lead to near-zero atomic distances, which leads to infinite potential energies. To enhance the numerical stability of the simulation, the distance computation between atoms was modified, and a constant offset of 0.001 Å was added to each interatomic distance for the computation of the Coulomb, Lennard–Jones, and GB terms. This small offset has negligible numerical effects in the low energy regions, but modifies the force field in the unphysical cases when atoms are clashing.

### Simulation protocol

All calculations were performed with the SIMONA^54^ code, which is available under https://www.int.kit.edu/nanosim/simona. The Trp-cage, Villin Headpiece and WW domain simulations were based on the pdb-files with codes 1L2Y^56^, 1VII^57^ and 2F21^58^, respectively. The force field parameters were assigned with the pdb2gmx program of Gromacs^112^. The structure was minimized with Gromacs^19^ and relaxed with backbone or sidechain moves using SIMONA at 50 K. The structure with the lowest energy was used as the reference for the native structure. We performed five to ten simulations each comprising 200 million MC-steps at different simulation temperatures, depending on the protein, i.e. 330–450 K, 360–460 K and 400–600 K for Trp-cage, Villin Headpiece and WW domain, respectively. An individual MC step comprises either a randomly selected backbone and sidechain dihedral rotation or a concerted move with equal probability. The angle change in the dihedral moves was drawn from a Gaussian distribution with a width of 18.3° for Villin Headpiece and 20° for Trp-cage and WW domain. In a “concerted move” a segment of 4 amino acids modified, changing all dihedral angles under the constraint that the endpoints of the segment do not change. In addition, rigid body rotations were applied by rotating the molecule around a random axis through its geometric center with a uniformly distributed rotation angle of up to 5°. The Metropolis acceptance criterion with Markov chain model was used to construct collective moves with the acceptance probability of 0.6 (60%), preserving detailed balance. The simulations operated at an effective time step of 260 fs/MC step, accelerating the sampling of the conformational space by about two orders of magnitude over all-atom explicit-solvent MD simulations. The first 10% of the steps of each simulation was discarded to permit equilibration. The elliplicity and CD spectra were computed with the analyses program CdPro^113^. Visualization of proteins was done using VMD^114^.

## Supplementary information

Supplementary information.
