# Supplementary material for: Sampling of the conformational landscape of small proteins with Monte Carlo methods
Source: Sci Rep. 2020 Oct 23;10:18211. doi: 10.1038/s41598-020-75239-7 (PMC7585447; doi:10.1038/s41598-020-75239-7)
Supplement: Supplementary file 1 — Supplementary information. [file 41598_2020_75239_MOESM1_ESM.pdf]

Supporting information for:

Sampling of the conformational landscape of small proteins with Monte Carlo methods

Nana Heilmann, Moritz Wolf, Mariana Kozlowska, Elaheh Sedghamiz, Julia Setzler, Martin Brieg, Wolfgang Wenzel

Institute of Nanotechnology (INT), Hermann-von-Helmholtz-Platz 1, 76344 Eggenstein-Leopoldshafen, Karlsruhe Institute of Technology (KIT), Germany

E-mail: wolfgang.wenzel@kit.edu

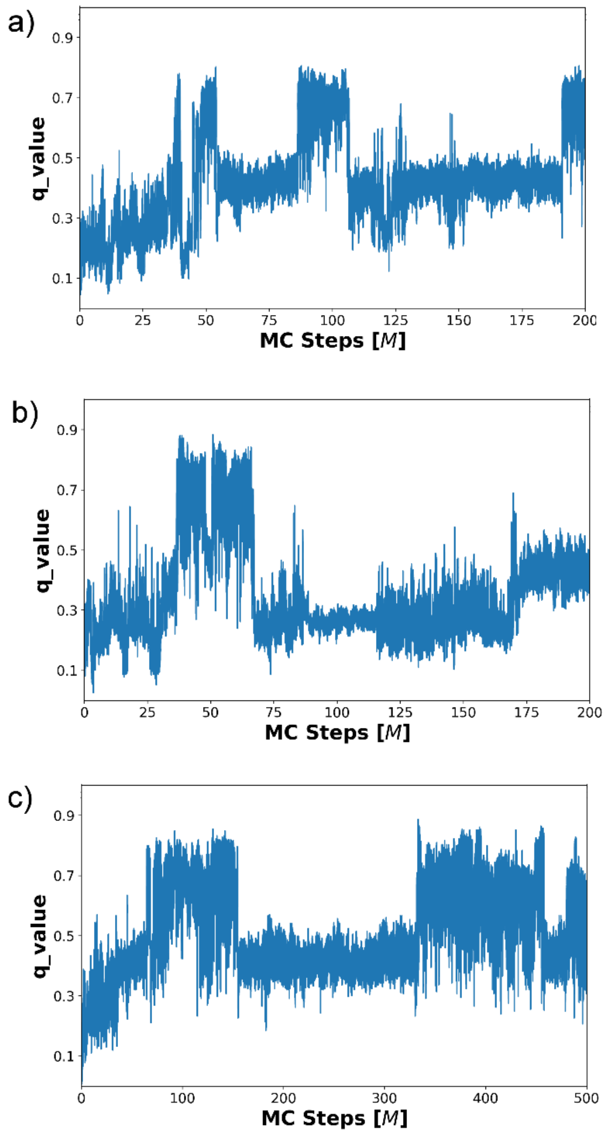

Figure S1. *MC simulations of the Trp-cage starting from unfolded protein. Selected MC trajectories at the transition temperature of 370 K showing the change of the fraction of native contacts. Multiple folding and unfolding events are observed. MC simulation started from*

unfolded Trp-cage structure with Q = 0.07 (shown in green in Figure 1 in the main body) (a) and Q = 0.12 (b, c).

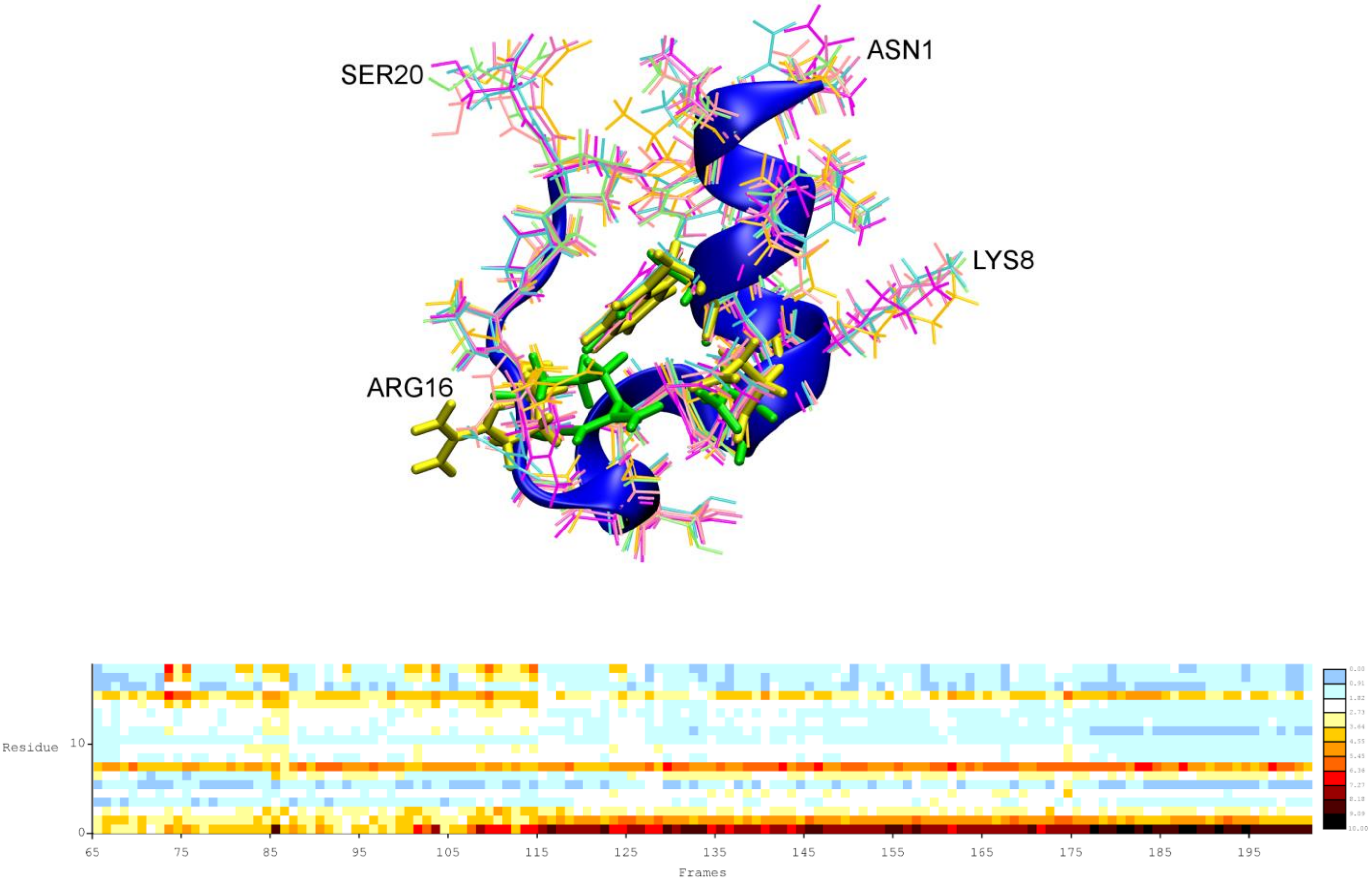

Figure S2. *Upper panel: Overlay of different refolded conformers of Trp-cage, obtained from the MC simulations at the folding temperature. The most flexible residues are marked with their names. Trp-6, Asp-9 and Arg-16 in the native structure are marked in green, while that residues in refolded state are in yellow. Bottom panel: Heatmap of particular RMSD residue change in different refolded conformers as a fluctuation from its position in the native form (changes close to the native-like structure of Trp-cage are in blue, while residues with high RMSD are in brown and dark brown). Visualization was done in VMD (version 1.9.2beta1) http://www.ks.uiuc.edu/Research/vmd/*

Native contacts, residue contact map, potential of mean force

Our definition of native contacts follows Noel et al[1], where all atoms are given a radius r=1 Å and the cut-off radius r_cutoff=6 Å for short range contacts is used. In the calculation of the φ-values a residue was considered native, when more than 70% of its native contacts are formed. The φ-value analysis was made with eSMBTools61. The potentials of mean force are defined as:

$$g(q) = -kTln(\rho(q))$$

where $\rho(q)\rho(q)$is the probability amplitude of the reaction coordinate.

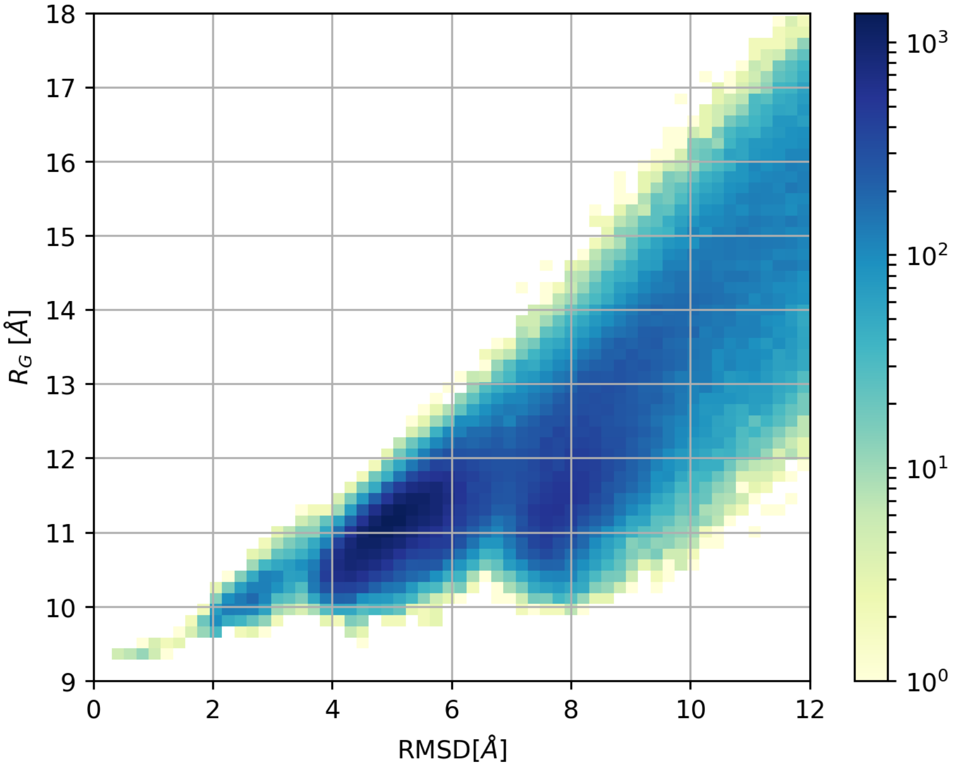

Figure S3: *Correlation between Free Energy as a function of RMSD and radius of gyration of 1VII.*

Temperature Calibration

In general, the usage of implicit solvation is connected to increase of the folding temperatures, in comparison to the values observed both in experiment and simulated in MD with explicit water. Here, we observe the same trend. We, therefore, computed the specific heat for the Villin headpiece as a function of temperature using WHAM58 (see Figure S3)[2]. Comparison with the experimentally determined specific heat, see Figure 2 in[3] suggested a temperature

shift of 83 K, which was applied in the analysis of the Villin headpiece. The shift between experimental and simulated temperature is also known in REMD simulations[4–6] and MD simulation with implicit solvent model[7]. This temperature shift of 10 to 50 K has been also observed in MD simulation with explicit solvent model and the same force field[4,8,9]. No attempt was made to modify the parameters of the implicit solvent model to capture the temperature dependence of solvation.

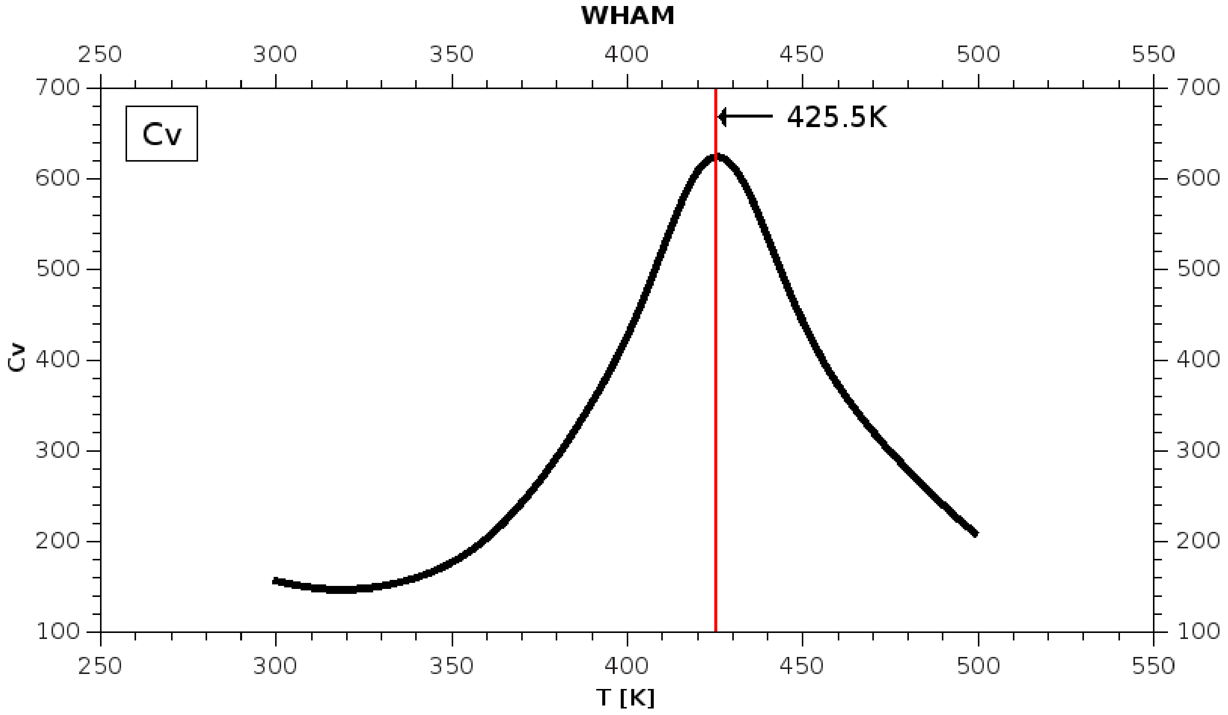

Figure S4: *Specific heat capacity (C_v), in kcal mol-1 K-1, for the Villin headpiece as a function of temperature. The temperature in the heat capacity maximum was used for temperature calibration of MC simulations of Villin headpiece.*

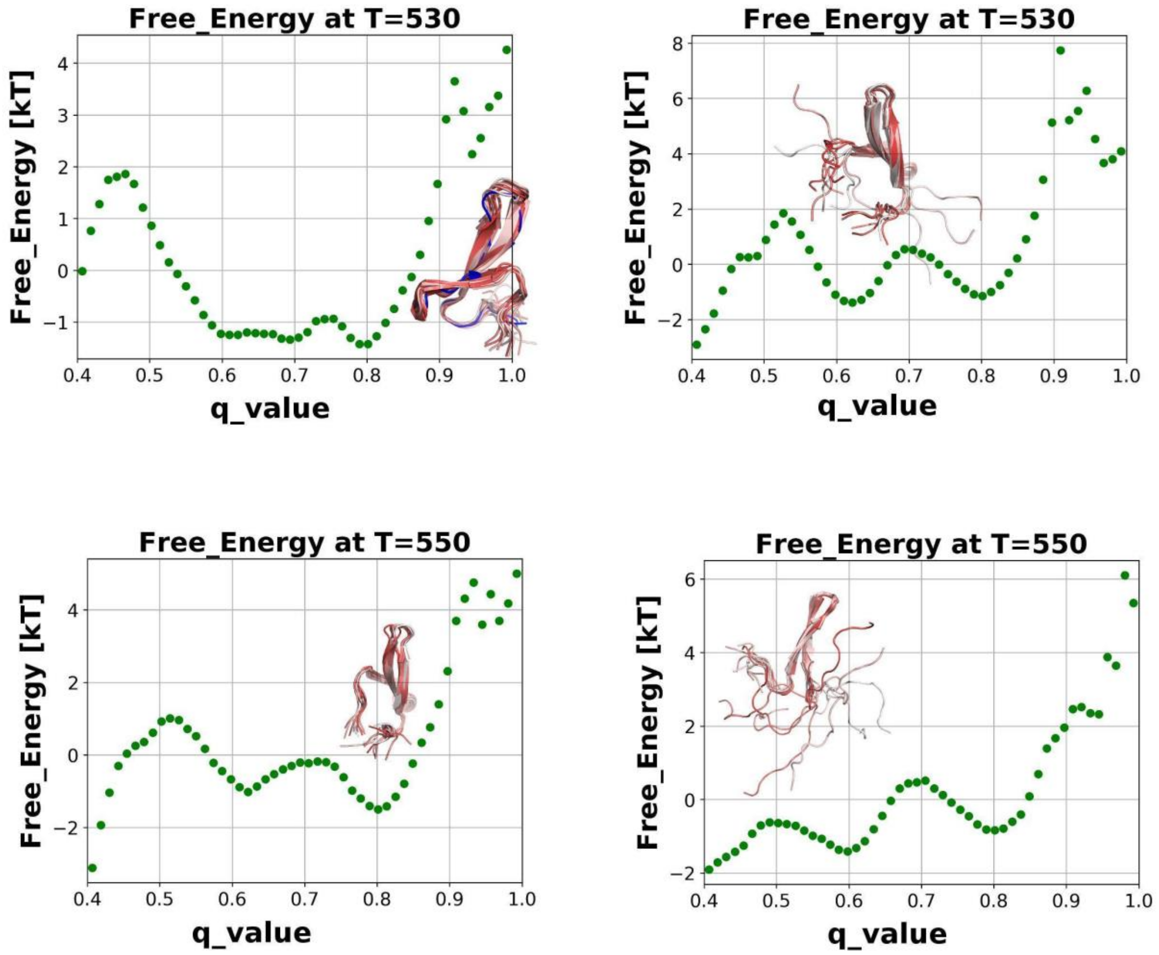

Figure S5. *MC simulations of the WW domain. Representative free energy plots as a function of the fraction of native contacts (q_value), representing different ensembles of the protein. The overlay of multiple conformers in the particular ensemble is depicted accordingly. Visualization was done in VMD (version 1.9.2beta1) http://www.ks.uiuc.edu/Research/vmd/*

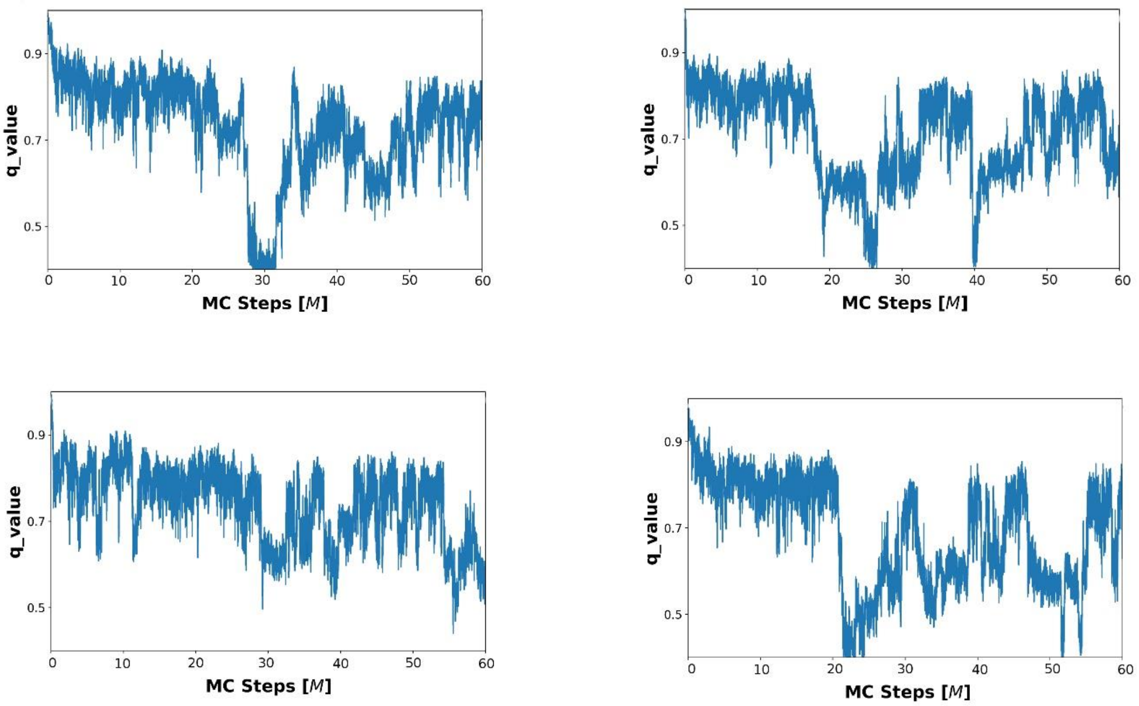

Figure S6: *MC simulations of the WW domain. Selected MC trajectories near the transition temperature of 530 - 550 K showing the change of the fraction of native contacts. Multiple folding and unfolding events are observed.*

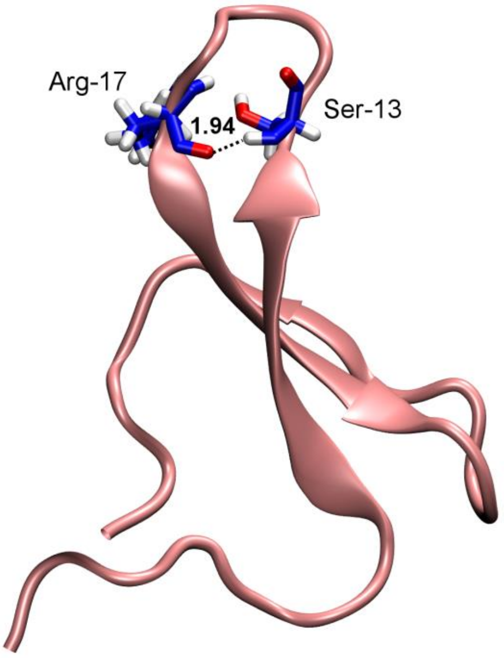

Figure S7: *Solvent exposed N-H···O hydrogen bond between Ser-13 and Arg-17 stabilizing the β-hairpin of WW domain (pdb structure 1L2Y).*

References

1. Noel, J. K., Whitford, P. C. & Onuchic, J. N. The Shadow Map: A General Contact Definition for Capturing the Dynamics of Biomolecular Folding and Function. *J. Phys. Chem. B* **116**, 8692–8702 (2012).
2. Noel, J. K. *et al.* SMOG 2: A Versatile Software Package for Generating Structure-Based Models. *PLoS Comput Biol* **12**, e1004794 (2016).
3. Kubelka, J., Henry, E. R., Cellmer, T., Hofrichter, J. & Eaton, W. A. Chemical, physical, and theoretical kinetics of an ultrafast folding protein. *Proceedings of the National Academy of Sciences* **105**, 18655–18662 (2008).
4. Zhou, R. Trp-cage: Folding free energy landscape in explicit water. *Proceedings of the National Academy of Sciences* **100**, 13280–13285 (2003).
5. Paschek, D., Nymeyer, H. & García, A. E. Replica exchange simulation of reversible folding/unfolding of the Trp-cage miniprotein in explicit solvent: On the structure and possible role of internal water. *Journal of Structural Biology* **157**, 524–533 (2007).
6. Beck, D. A. C., White, G. W. N. & Daggett, V. Exploring the energy landscape of protein folding using replica-exchange and conventional molecular dynamics simulations. *Journal of Structural Biology* **157**, 514–523 (2007).
7. Shao, Q. & Zhu, W. Assessing AMBER force fields for protein folding in an implicit solvent. *Phys. Chem. Chem. Phys.* **20**, 7206–7216 (2018).
8. Robustelli, P., Piana, S. & Shaw, D. E. Developing a molecular dynamics force field for both folded and disordered protein states. *Proc Natl Acad Sci USA* **115**, E4758–E4766 (2018).
9. Snow, C. D., Zagrovic, B. & Pande, V. S. The Trp Cage: Folding Kinetics and Unfolded State Topology via Molecular Dynamics Simulations. *J. Am. Chem. Soc.* **124**, 14548–14549 (2002).
